# Chronic Intermittent Ethanol and Withdrawal Suppress Evoked and Spontaneous GABA Release Onto Distinct Populations of Basolateral Amygdala Principal Neurons

**DOI:** 10.1111/adb.70080

**Published:** 2025-09-21

**Authors:** Michaela E. Price, Hailey X. Egido‐Betancourt, Sarah E. Sizer, Brian P. Parrish, Nancy J. Alexander, Kimberly F. Raab‐Graham, Brian A. McCool

**Affiliations:** ^1^ Department of Translational Neuroscience Wake Forest University School of Medicine Winston‐Salem North Carolina USA

**Keywords:** feed‐back inhibition, feed‐forward inhibition, opioid receptors, whole‐cell voltage‐clamp electrophysiology

## Abstract

Unique populations of basolateral amygdala (BLA) neurons regulate anxiety and reward through projections targeting downstream regions like the bed nucleus of the stria terminalis (BNST) and nucleus accumbens (NAC). We showed previously that withdrawal from chronic ethanol exposure (CIE/WD) produced population‐ and sex‐specific alterations to distinct glutamatergic inputs. The current study examined GABAergic function in these distinct populations (BLA^NAC^ and BLA^BNST^ neurons). We found that CIE/WD diminished feed‐forward GABA release from lateral paracapsular cells (LPCs) specifically onto male BLA^NAC^ neurons. Pharmacological manipulations showed this dysregulation was caused by the enhanced activity of μ‐opioid receptors. CIE/WD did not alter evoked GABA release from local interneurons onto either population. However, females expressed greater GABA release from these local interneurons compared to males. Immunostaining and confocal microscopy revealed lower colocalization between the GABA vesicular transporter, vGAT and parvalbumin in females, indicating that greater GABA releases from local interneurons in this sex may be a compensatory response to lower levels of perisomatic innervation by PV^+^ interneurons. Consistent with this, there were no sex differences related to spontaneous GABAergic synaptic events although CIE/WD decreased their frequency specifically in BLA^BNST^ neurons from both sexes. Altogether, these findings demonstrate that CIE/WD dynamically alters GABAergic function in an input‐, sex‐ and population‐specific fashion. Moreover, there are basal sex differences in both the anatomy of BLA GABAergic synapses and their function.

## Graphical abstract

Rat basolateral amygdala principal neurons projecting to the nucleus accumbens (BLA^NAc^) and bed nucleus of the stria terminalis (BLAB^NST^) represent independent cell populations. In male BLA^NAc^ neurons only (left), CIE/WD decreases feedforward GABA release from lateral paracapsular interneurons. In BLA^BNST^ neurons (right), GABA release from ‘local’ feedback interneurons is greater in females; but CIE/WD diminishes spontaneous GABA release attributable to these ‘local’ interneurons in both sexes. Thus, CIE/WD dysregulation of BLA GABAergic neurotransmission is sex‐, synapse‐ and population‐specific.

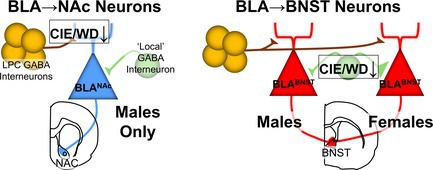

## 1 Introduction

Withdrawal‐induced anxiety and excessive alcohol consumption are hallmarks of alcohol use disorder [1]. Rodent models of alcohol dependence like the chronic intermittent ethanol (CIE) paradigm have been used to understand the neurobiological mechanisms and circuits driving withdrawal‐induced pathologies like withdrawal‐associated anxiety and excessive alcohol consumption [2, 3, 4, 5, 6]. The basolateral amygdala (BLA) is one of several brain regions that regulate anxiety and reward‐related behaviours, and it does so through its downstream projections. For instance, activating the BLA projection to the nucleus accumbens (NAC) core promotes positive reinforcement, the consumption of rewarding substances like sucrose and the reinstatement of drug‐seeking [7, 8]. The NAC core has also been implicated in alcohol‐related behaviours, including the reinstatement of alcohol‐seeking, binge drinking and alcohol‐conditioned place preference [9, 10, 11]. In contrast, the BLA modulates anxiety‐like behaviours [12, 13] as does activating its downstream targets like the BNST [14, 15, 16]. Glutamatergic principal neurons drive BLA output to downstream brain regions and account for approximately 85% of the neurons in this brain region [17]. Therefore, understanding how CIE/WD dysregulates BLA principal neurons that project to regions regulating anxiety‐like behaviours and alcohol consumption (e.g., BNST and NAC, respectively) will present more focused targets for AUD treatments.

At least two anatomically distinct populations of GABAergic interneurons regulate the activity of BLA principal neurons: ‘local’ interneurons and lateral paracapsular cells (LPCs). ‘Local’ interneurons are dispersed throughout the BLA, whereas LPCs are clustered along the external capsule bordering the lateral side of the BLA [18]. LPCs and local interneurons differ with respect to the expression of calcium‐binding proteins, neuropeptides, subcellular projection targets and various neurotransmitter receptors. For example, LPCs uniquely express dopamine D1 and μ‐opioid receptors [19, 20] and provide substantial feed‐forward inhibition to BLA principal neurons [18]. Local interneurons are highly diverse and express one or more calcium‐binding proteins or neuropeptides, including parvalbumin (PV), calbindin, calretinin, neuropeptide Y, cholecystokinin and somatostatin [18, 21, 22]. These neurons provide substantial feedback‐type inhibition by sending robust synaptic inputs onto BLA principal neuron perisomatic regions [23].

We previously showed that CIE/WD reduces GABA release from LPCs onto BLA principal neurons in male rats but did not affect GABA release from local interneurons [24, 25]. Importantly, a previous study also demonstrated that CIE/WD differentially regulates glutamatergic projections onto BLA neurons projecting to the NAC (BLA^NAC^ cells) and the BNST (BLA^BNST^). CIE/WD increased postsynaptic glutamatergic function expressed by male but not female BLA^NAC^ neurons and increased presynaptic function in BLA^BNST^ neurons in both sexes [5]. Given that there are sex differences in inhibitory function in the BLA [26], it is unclear if GABA neurotransmission onto both neuronal populations will be impacted by the CIE/WD or if CIE effects on female interneurons will mirror those found in males. Given that the NAC and BNST are downstream targets of the BLA and regulate reward‐ and anxiety‐like behaviours, dysregulation of GABAergic inhibition onto BLA^NAC^ and BLA^BNST^ neurons would influence the expression of behavioural pathologies following ethanol dependence.

## 2 Methods

### 2.1 Animals

Male and female Sprague–Dawley rats were obtained from Envigo (Indianapolis, IN) and were housed separately. Upon arrival, all rats were pair‐housed and maintained on a reverse 12‐h light–dark cycle (9 AM lights off, 9 PM lights on). The rats were provided water and rat chow ad libitum for the entire study. Rats acclimated to the animal housing rooms for at least 48 h prior to undergoing stereotaxic surgeries at approximately 5 weeks of age (120–150 g). Animal care procedures followed the National Institutes of Health's *Guide for the Care and Use of Laboratory Animals* and were approved by the Wake Forest Animal Care and Use Committee (WF‐ACUC).

### 2.2 Stereotaxic Surgeries

**FIGURE 1:**
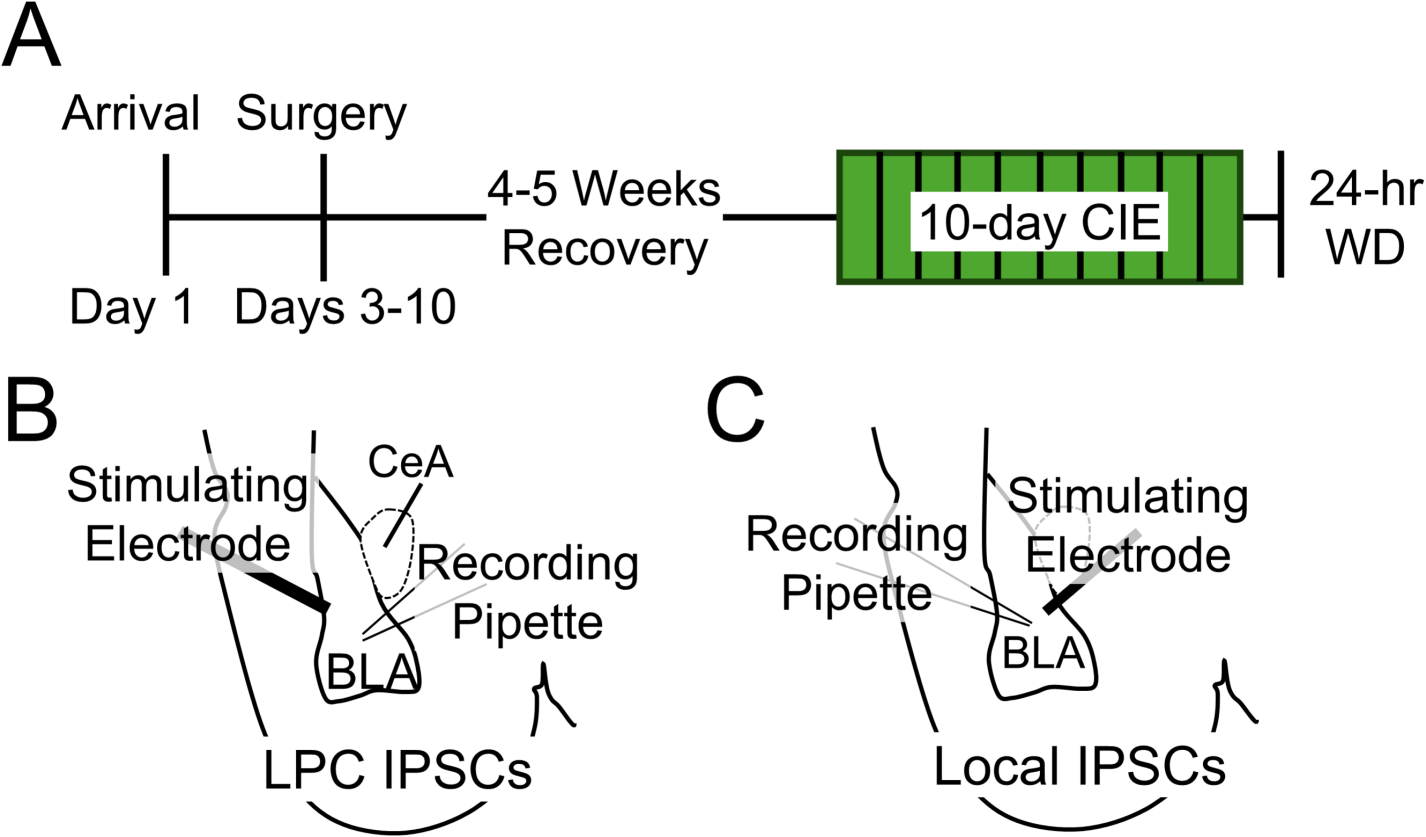
Experimental design*.* (A) Experimental timeline. (B) Diagram of recording evoked IPSCs from LPCs. The stimulating electrode is placed within the external capsule, where LPCs are clustered. (C) Diagram of recording evoked IPSCs from ‘local’ interneurons. The stimulating electrode was placed within the BLA, medial to the recording site.

The experimental timeline is depicted in Figure 1A. Projection‐specific populations of BLA neurons were labelled using retrogradely transported adeno‐associated viral vectors (AAVrg) microinjected into downstream terminal fields during stereotaxic microinjection surgeries, as previously described [5]. Briefly, rats were anaesthetized using 3% isoflurane and maintained under continuous 2%–3% isoflurane during the surgery with the oxygen flowmeter set at a rate of 1.0 L/min. The rats received a bilateral microinjection of an AAVrg encoding GFP (AAVrg‐CAG‐GFP; Addgene 37 825) or tdTomato (AAVrg‐CAG‐tdTomato; Addgene 59 642) into either the NAC or the BNST using the following coordinates (millimetres in relation to bregma): NAC (AP +1.80, ML ± 1.50, DV 6.72) and BNST (AP −0.36, ML ± 0.80, DV 6.45). A syringe pump microinjected the AAVrg at a rate of 0.1 μL/min over 5 min (total volume: 0.5 μL per side), and the injectors were left in place for 5–10 min to allow for virus diffusion into the NAC or BNST. At the end of the surgery, rats were sutured and received meloxicam (1 mg/kg, sc, Patterson Veterinary Supply) for analgesia and warmed sterile saline (2 mL, sc). The rats were then single‐housed following the surgery and received additional meloxicam doses (1 mg/kg, sc) for the next 2 days. We removed sutures 7 days after the surgery, at which point the rats were pair‐housed for the remainder of the experiment. We confirmed viral expression patterns 6–7 weeks after the microinjection surgery using fluorescent microscopy of coronal brain slices containing the NAC and BNST and excluded rats with incorrect viral placement.

### 2.3 CIE/WD

Approximately 4 weeks after the viral microinjection surgery, pair‐housed rats were exposed to CIE via vapour inhalation for 10 consecutive days using our standard lab procedure (Figure 1A; [5, 13, 27, 28, 29]). Briefly, rats in their home cages were placed into large, custom‐built Plexiglas vapour chambers (Triad Plastics, Winston‐Salem, NC). Ethanol vapour was produced by pumping compressed air through a bubbler stone submerged in 95% ethanol. The ethanol vapour was pumped into the vapour chamber at a constant rate (16 L/min) and maintained at 15–25 mg/L for 12 h/day during the light cycle (beginning at 9 PM). Age‐matched, air‐exposed (AIR) control rats were housed similarly to CIE/WD‐exposed rats but received only room air. Blood samples (tail vein) were taken at least once during the 10‐day ethanol vapour exposure to monitor blood ethanol concentrations (BECs) and to adjust ethanol vapour levels when necessary. BECs were measured using a commercially available alcohol dehydrogenase/NADH enzymatic assay (Carolina Liquid Chemistries). The average BECs during the CIE exposure were 212 ± 7.0 mg/dL in males (*n* = 35) and 223.0 ± 6.7 mg/dL in females (*n* = 29; *t* = 1.048, *p* = 0.299, *t*‐test). The rats were removed from the Plexiglas chambers after the final ethanol vapour exposure for a 24‐h withdrawal period prior to electrophysiological recordings or immunohistochemistry. This 10‐day CIE paradigm has consistently produced anxiety‐like behaviour during withdrawal in both male and female rats [30], increased alcohol intake in male rats [30] and produced neurophysiological changes in the BLA in both sexes [5, 13, 27, 28, 29].

### 2.4 Electrophysiology

#### 2.4.1 Slice Preparation

The rats were anaesthetized with isoflurane prior to decapitation, in accordance with animal care protocols approved by WF‐ACUC. The brains were rapidly removed and incubated for 5 min in an ice‐cold sucrose‐modified artificial cerebrospinal fluid (aCSF) solution that was equilibrated with 95% O_2_ and 5% CO_2_ and contained the following (in millimolars): 180 sucrose, 30 NaCl, 4.5 KCl, 1 MgCl_2_·6H_2_O, 26 NaHCO_3_, 1.2 NaH_2_PO_4_, 10 ᴅ‐glucose and 0.10 ketamine. Coronal BLA slices (250 μm) were prepared in oxygenated, ice‐cold sucrose‐modified aCSF using a VT1200/S vibrating blade microtome (Leica). The slices were transferred to oxygenated, standard aCSF solution (28°C) containing the following (in millimolars): 126 NaCl, 3 KCl, 1.25 NaH_2_PO_4_, 2 MgSO_4_·7H_2_O, 26 NaHCO_3_, 10 ᴅ‐glucose and 2 CaCl_2_·2H_2_O. The slices were incubated in oxygenated, standard aCSF solution for at least 1 h to recover until electrophysiological recordings were performed. All chemicals were obtained from Tocris (Ellisville, MO) or Sigma‐Aldrich (St. Louis, MO).

#### 2.4.2 Whole‐Cell Patch‐Clamp Electrophysiology

We placed BLA slices in a submersion‐type recording chamber and continuously perfused them with oxygenated, room‐temperature standard aCSF at a rate of ~2 mL/min. We used an Olympus BX51WI infrared differential interference contrast (IR‐DIC) microscope with fluorescence attachments to identify GFP‐ or tdTomato‐expressing BLA^NAC^ neurons and BLA^BNST^ neurons. The fluorescent neurons were confirmed to be principal glutamatergic neurons by using membrane capacitance (> 100 pF) measured from hyperpolarizing (−5 mV) steps in each recording. Recordings included for analysis also had low access resistance (≤ 25 MΩ). Whole‐cell voltage‐clamp recordings were performed using recording electrodes filled with a Cs‐gluconate intracellular solution containing the following (in millimolars): 145 CsOH, 10 EGTA, 10 HEPES, 5 NaCl, 1 MgCl_2_·6H_2_O, 4 Mg‐ATP, 0.4 Na‐GTP, 0.4 QX314‐Cl and 1 CaCl_2_·2H_2_O. The pH of the intracellular solution was adjusted to ~7.25–7.35 pH with ᴅ‐gluconic acid; and the osmolarity was adjusted to ~280–290 mOsm/L with sucrose. We pharmacologically isolated GABAergic inhibitory postsynaptic currents (IPSCs) using APV (50 μM, NMDA receptor antagonist) and DNQX (20 μM, AMPA receptor antagonist) in the extracellular aCSF. We recorded IPSCs at a membrane holding potential of −10 mV. We excluded cells from analysis if their holding currents or access resistance changed by ≥ 20% during the recordings.

In some experiments, GABAergic synaptic responses were electrically evoked every 30 s with a platinum/iridium concentric bipolar stimulating electrode (FHC Inc., Bowdoin, ME). The stimulating electrode was placed either within the external capsule to stimulate GABA release from LPCs (Figure 1B) or within the BLA, just medial to the recording site, to stimulate GABAergic responses mediated by ‘local’ interneurons (Figure 1C, [24, 31]). Stimulus intensities were submaximal and normalized across cells to elicit IPSCs with amplitudes of ~100 pA. To measure apparent GABA release, we delivered paired electrical stimuli of equal intensity at a 50‐ms inter‐stimulus interval to the external capsule (LPCs) or within the BLA (‘local’ interneurons). Paired‐pulse ratios (PPRs) at these short inter‐stimulus intervals are inversely related to presynaptic release probability [32]. The PPR was calculated using the amplitude of the two electrically evoked IPSCs (PPR = Peak 2 Amplitude/Peak 1 Amplitude). The average PPR was determined from a 5‐min recording. We used a syringe pump to acutely deliver DAMGO (1 μM, Tocris) at 0.1 mL/min from a 20X stock in aCSF. For some experiments, we also recorded spontaneous IPSCs (sIPSCs). In the BLA, these events primarily reflect the synaptic activity of ‘local’ interneurons [33]. Data were acquired at 5 kHz and low‐pass‐filtered at 2 kHz using an Axopatch 700B amplifier and pCLAMP 10 software (Molecular Devices). We analysed sIPSC data using MiniAnalysis (Synaptosoft) and determined median amplitude and frequency values for each individual cell prior to averaging within groups [12, 24].

### 2.5 Immunohistochemistry and Confocal Microscopy

Air‐ and CIE‐exposed rats (*n* = 3 from each sex and treatment) were transcardially perfused with ice‐cold PBS and post‐fixed with 4% *p*‐formaldehyde overnight. Coronal BLA sections that are 25 μm thick were taken on a sliding microtome (Leica SM, 2010F) and placed in cryoprotectant (15% ethylene glycol, 10% glycerol, 0.05 M PBS; [34]). BLA sections (*n* = 8 from each group) were permeabilized, blocked (0.4% Triton, 10% normal donkey serum or normal goat serum, 0.25% Tween 20) and immunostained overnight at 4°C with the following antibodies: chicken anti‐MAP 2 (Invitrogen, PA1‐10005; 1:1000), rabbit anti‐vGAT (SySy, Cat. No. 13‐002; 1:500) and mouse anti‐PV (Sigma, P3088; 1:1000). Following primary incubation, sections were washed with 1XPBS three times and incubated with secondary antibodies conjugated to Alexa Fluor 405 (donkey anti‐chicken, Code: 703‐475‐155 or goat anti‐chicken, ab175674, Abcam), Alexa Fluor 488 (donkey anti‐mouse, 715‐545‐150 or goat‐anti‐mouse, 115‐545‐003; Jackson ImmunoResearch Laboratories) and Alexa Fluor 647 (donkey anti‐rabbit, A31573, Thermo Fisher Scientific; or goat anti‐rabbit, A21245; Invitrogen) at 1:400 for 4 h at room temperature. Slices were mounted (Aqua‐Poly/Mount, 18 606‐20, Polysciences), and all images were acquired and analysed using the same settings on a Nikon A1 plus confocal microscope. BLA slices were imaged with a 60×/1.40 oil Nikon Plan Apo immersion objective, at 1024 × 1024 pixels, with sequential scanning. A 10‐μm z‐stack was collected at 1‐μm optical sections containing tdTomato^+^ neurons. The experimenter was blind to specific treatments until the statistical analysis. Images were analysed in the Nikon NIS Elements AR (Version 4.40.00) software package. A region of interest (ROI) was drawn around each tdTomato^+^ soma, and both relative puncta intensity measures and Mander's overlap coefficients between vGAT and PV channels were collected from each region as described [35, 36]. Data across individual ROIs (~70 neurons per brain slice) were averaged within each slice (*n* = 8 per condition from three animals or two to three slices per animal) and used for statistical analysis.

### 2.6 Data Analysis

In all experiments, data from BLA^NAC^ neurons and BLA^BNST^ neurons were analysed separately. Statistical analyses were conducted using Prism software (GraphPad). Differences between groups were analysed using two‐way ANOVAs with CIE/WD and sex as main factors. Where interactions occurred between these factors, Bonferroni post hoc tests were used to determine the locus of the effect. The value of *p* < 0.05 was considered statistically significant. All data are presented as mean ± SEM.

## 3 Results

### 3.1 CIE/WD Reduces Evoked LPC GABA Release Onto Male BLA^NAc^ Neurons

**FIGURE 2:**
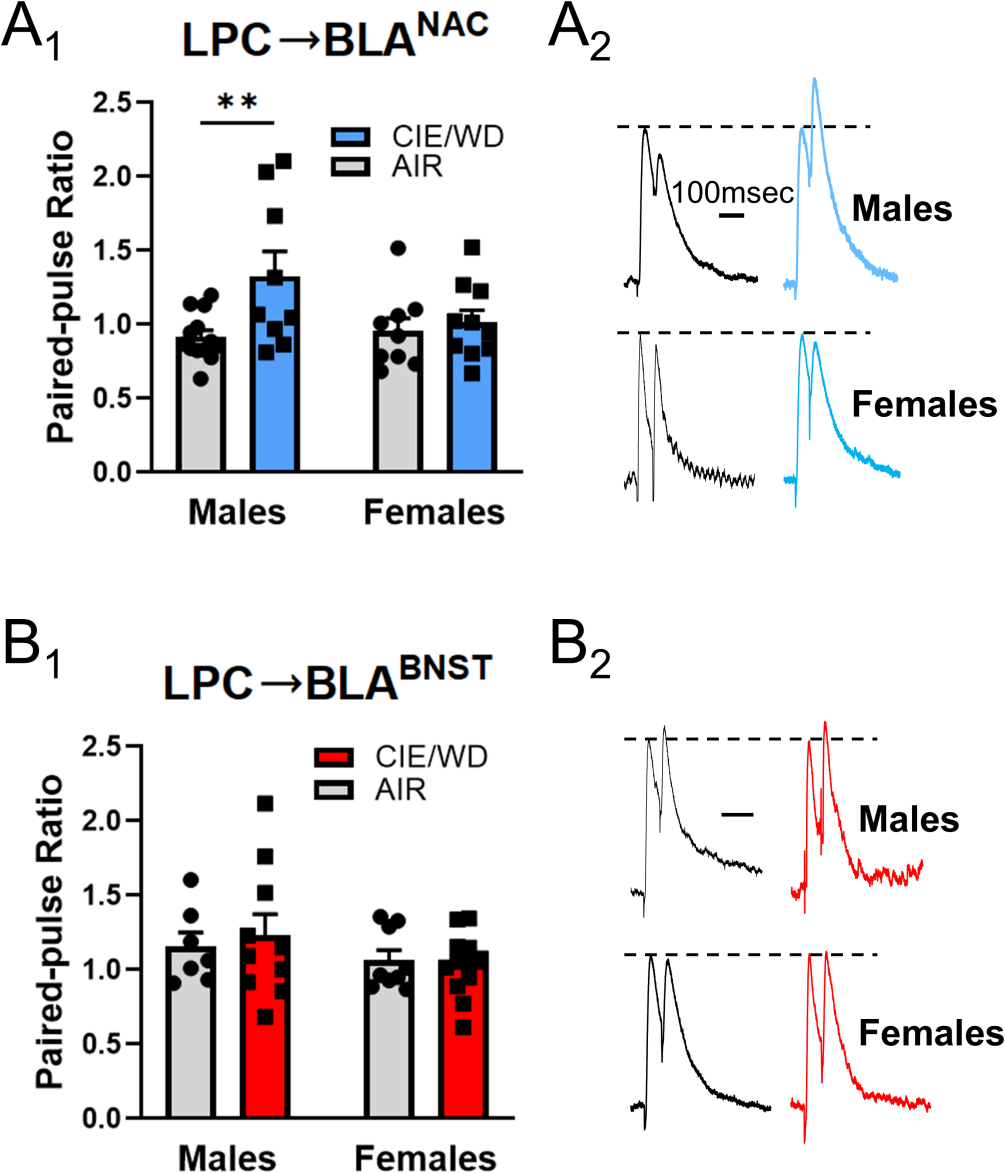
CIE/WD reduces GABA release from LPCs onto male BLA^NAC^ neurons*.* (A) PPR at LPC synapses with BLA^NAC^ neurons. A two‐way ANOVA revealed (A_1_) a significant main effect of CIE/WD (*p < 0.05) and a trending interaction effect between CIE/WD and sex (*p* = 0.077). CIE/WD increased the PPR (decreased GABA release) at LPC synapses with BLA^NAC^ neurons in males (*N* = 13 AIR, 9 CIE/WD, Bonferroni post hoc test *p* < 0.01**), but not females (*N* = 10 AIR, 10 CIE/WD, Bonferroni post hoc test *p* > 0.99). Representative traces (A_2_) of paired‐pulse responses at LPC synapses with BLA^NAC^ neurons; trace amplitudes have been normalized to the amplitude of the first IPSC to emphasize changes in the PPR. (B) PPR at LPC synapses with BLA^BNST^ neurons. A two‐way ANOVA (B_1_) indicated no significant effects on the PPR at LPC synapses with BLA^BNST^ neurons (males *N* = 7 AIR, 10 CIE/WD; females *N* = 10 AIR, 12 CIE/WD). Representative traces (B_2_) of paired‐pulse responses at LPC synapses with BLA^BNST^ neurons.

CIE/WD decreases GABA release probability from LPC synapses onto a general population of male BLA principal neurons [24, 25], suggesting ethanol dependence disrupts a critical source of feed‐forward inhibition for these cells. Importantly, reward‐ and avoidance‐like behaviours are regulated in part by distinct populations of BLA principal neurons projecting to downstream regions like the NAC and BNST [7, 8, 16, 37, 38, 39]. We therefore measured the impact of CIE/WD on GABA release from LPCs onto BLA^NAC^ neurons or BLA^BNST^ neurons in both male and female rats. At LPC ➔ BLA^NAC^ synapses (Figure 2A), a two‐way ANOVA using CIE/WD and sex as main factors revealed a trending CIE/WD × sex interaction effect, *F*(1, 37) = 3.292, *p* = 0.078, and a significant main effect of CIE/WD, *F*(1, 37) = 5.953, *p = 0.019, and on the PPR. A Bonferroni post hoc test found that CIE/WD significantly increased the PPR in male rats (**p = 0.007) but had no effect in females (*p* > 0.999). In contrast, for LPC ➔ BLA^BNST^ synapses, there was no interaction or significant impact of CIE/WD or sex on the PPR (Figure 2B). Altogether, these data show that CIE/WD selectively decreases GABA release from LPCs onto BLA^NAC^ neurons in male rats.

### 3.2 μ‐Opioid Receptors Govern Decreased GABA Release at Male LPC–BLA^NAC^ Synapses

**FIGURE 3:**
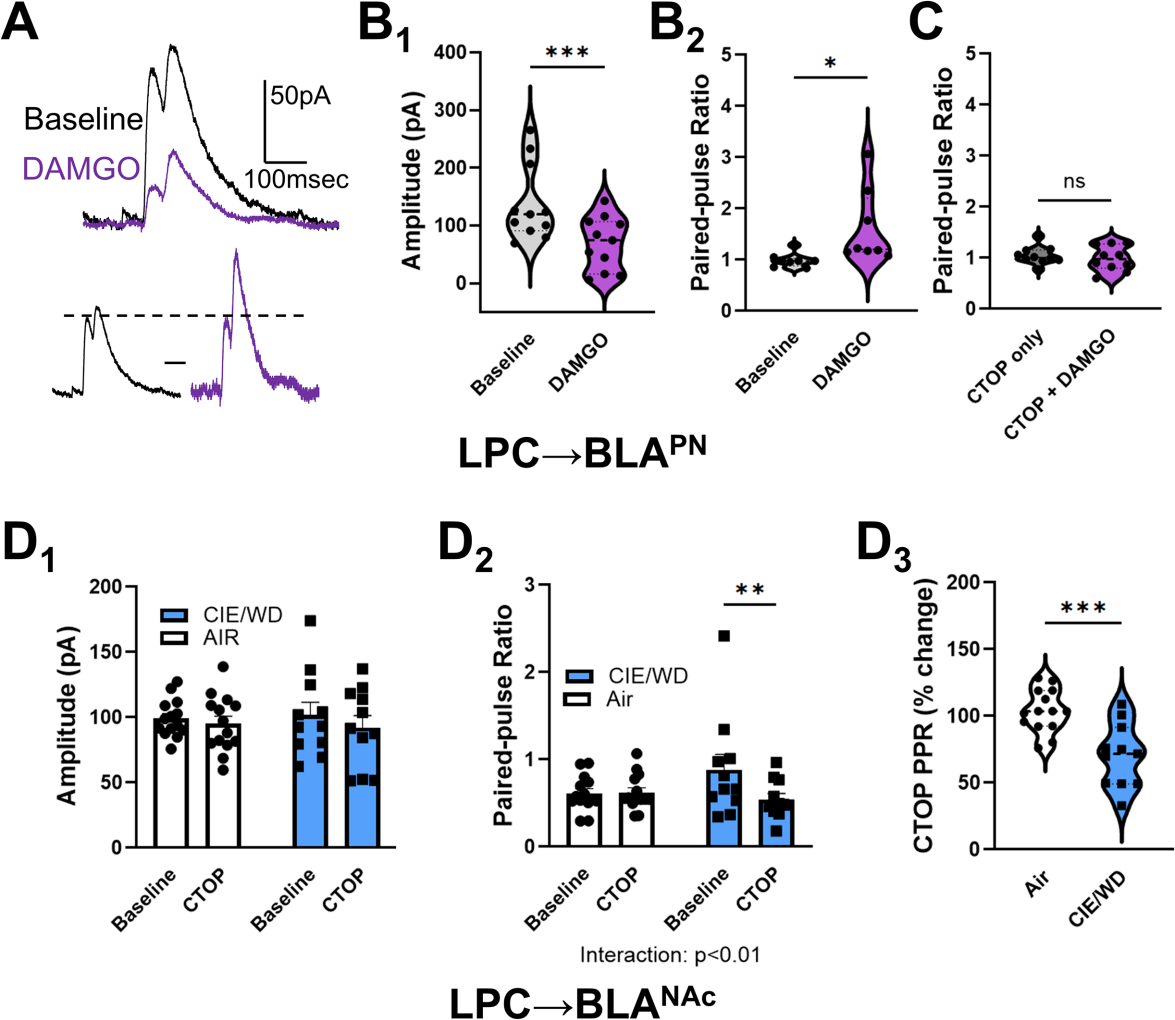
CIE/WD enhances mu receptor inhibition of LPC GABAergic synapses onto BLA^NAc^ neurons*.* (A) The mu receptor agonist DAMGO (1 μM) substantially suppressed LPC GABA IPSCs recorded from a general population of BLA principal neurons (BLA^PN^). (B) DAMGO significantly suppressed the LPC IPSC pulse 1 amplitude (B_1_) and significantly increased the paired‐pulse ratio (B_2_), suggesting that DAMGO decreases GABA release from LPC synapses. (C) DAMGO inhibition of LPC synapses is blocked by the mu receptor antagonist CTOP (1 μM). (D) The mu receptor antagonist CTOP (1 μM) does not alter LPC GABA pulse 1 amplitude (D_1_) but significantly decreases PPR (increases release, D_2_) in CIE/WD‐treated male BLA^NAc^ neurons. Two‐way, repeated‐measures ANOVA with CIE/WD and CTOP as main factors. **p < 0.01 Bonferroni post hoc. CTOP effects expressed as percentage of change from baseline (D_3_) illustrate significant differences between air and CIE/WD samples. ***p < 0.001, unpaired *t*‐test.

LPCs express the μ‐opioid receptor and receive input from enkephalin‐immunoreactive terminals [20, 40]. μ receptor agonists selectively hyperpolarize the developmentally related medial/main intercalated GABAergic cells without directly influencing the excitability of BLA principal neurons [41]. In a general population of male BLA principal neurons (projection target not defined) from ethanol‐naïve animals, we found that the μ agonist [D‐Ala(2), N‐Me‐Phe(4), Gly(5)‐ol]‐enkephalin (DAMGO, 1 μM) significantly suppressed the LPC IPSC amplitude (*n* = 11, *t* = 4.715, ***p = 0.001, paired *t*‐test) and increased the PPR of these responses in a majority of these cells (8/11 neurons, *t* = 2.710, *p = 0.03, paired *t*‐test; Figure 3A,B). In a separate experiment, we included the high‐affinity μ receptor antagonist D‐Phe‐Cys‐Tyr‐D‐Trp‐Orn‐Thr‐Pen‐Thr‐NH_2_ (CTOP, 1 μM) in the extracellular solution and found that it blocked the effects of DAMGO on both LPC IPSC PPR (*t* = 0.5381, *p* = 0.604, paired *t*‐test; Figure 3C) and amplitude (not shown, CTOP only: 96.4 ± 7.0 pA, CTOP + DAMGO: 85.43 ± 7.8 pA, *t* = 1.266, *p* = 0.241, paired *t*‐test).

Using unidentified BLA principal neurons from CIE/WD‐exposed male rats, we tested the acute effects of the high‐affinity μ receptor antagonist D‐Phe‐Cys‐Tyr‐D‐Trp‐Orn‐Thr‐Pen‐Thr‐NH_2_ (CTOP, 1 μM) on LPC PPR. In four of 13 cells, CTOP produced negligible effects on LPC PPR (< 5% change). However, in the remaining nine cells, CTOP significantly reduced the LPC PPR from 1.45 ± 0.23 at baseline to 1.15 ± 0.16 (*t* = 3.967, **p = 0.004, paired *t*‐test). We next tested CTOP on male LPC → BLA^NAC^ synapses and found that it suppressed LPC PPR (increased release) in CIE/WD‐treated males but not AIR controls (Figure 3D_2_), CTOP × treatment interaction: *F*(1, 23) = 7.778, *p* = 0.01; Bonferroni's post‐test, **p = 0.003. These data together strongly suggest that CIE/WD enhances the function of μ opioid receptors which decrease GABA release at male LPC → BLA^NAC^ synapses.

### 3.3 Basal Sex Differences in Evoked GABA Release From ‘Local’ Interneurons

**FIGURE 4:**
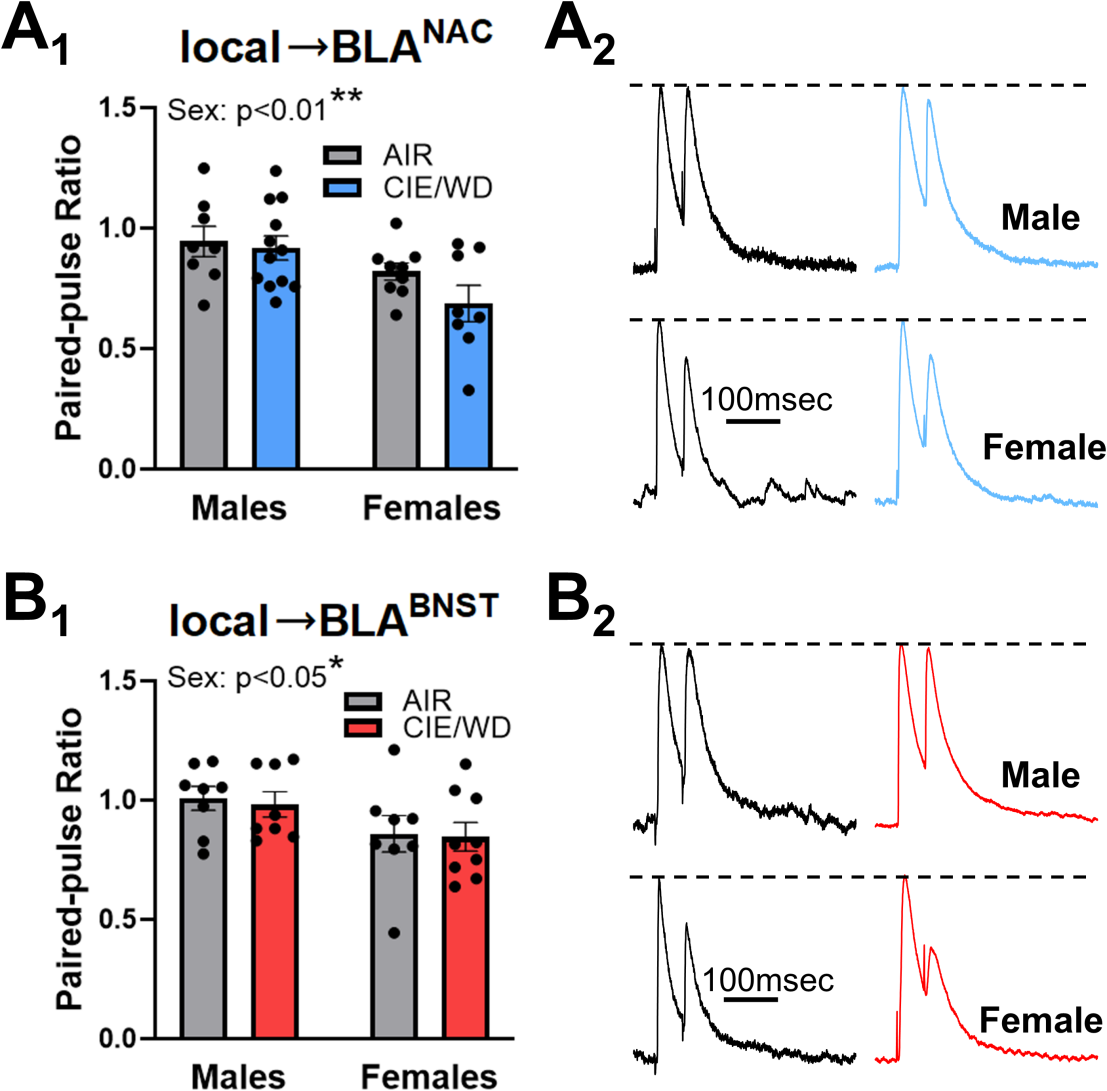
Basal sex differences in evoked GABA release from ‘local’ interneurons. (A) PPR at local interneuron synapses with BLA^NAC^ neurons. A two‐way ANOVA (A_1_) revealed a significant main effect of sex (**p < 0.01). Females (*N* = 8 AIR, 12 CIE/WD) had a lower PPR (higher GABA release, A_2_) at local interneuron synapses with BLA^NAC^ neurons than males (*N* = 9 AIR, 8 CIE/WD). (B) PPR at local interneuron synapses with BLA^BNST^ neurons. A two‐way ANOVA (B_1_) indicated a main effect of sex (*p < 0.05). Females (*N* = 8 AIR, 9 CIE/WD) had a lower PPR (higher GABA release, B_2_) at local synapses with BLA^BNST^ neurons compared to males (*N* = 8 AIR, 8 CIE/WD). All traces normalized to Peak 1 amplitude (dashed lines) to emphasize differences in PPR.

CIE/WD did not alter stimulated GABA release probability at ‘local’ interneuron synapses in a previous study with male rats [24, 25]; however, those studies did not include female rats and did not distinguish between various populations of BLA neurons defined by their projection targets. Therefore, we examined whether CIE/WD or sex altered evoked ‘local’ GABA release onto BLA^NAC^ and BLA^BNST^ neurons. At local interneuron synapses, PPR was lower in female rats compared to males in both BLA^NAC^ and BLA^BNST^ cells (Figure 4), BLA^NAC^, *F*(1, 33) = 9.712, **p = 0.004; BLA^BNST^, *F*(1,29) = 5.468, *p = 0.027, but there was no significant effect of CIE/WD and no interaction between sex and treatment in either population. This suggests that female local interneurons release more GABA onto principal neurons from both populations compared to males.

### 3.4 Females Have Lower Colocalization Between vGAT and PV Around BLA^NAC^ Somata

**FIGURE 5:**
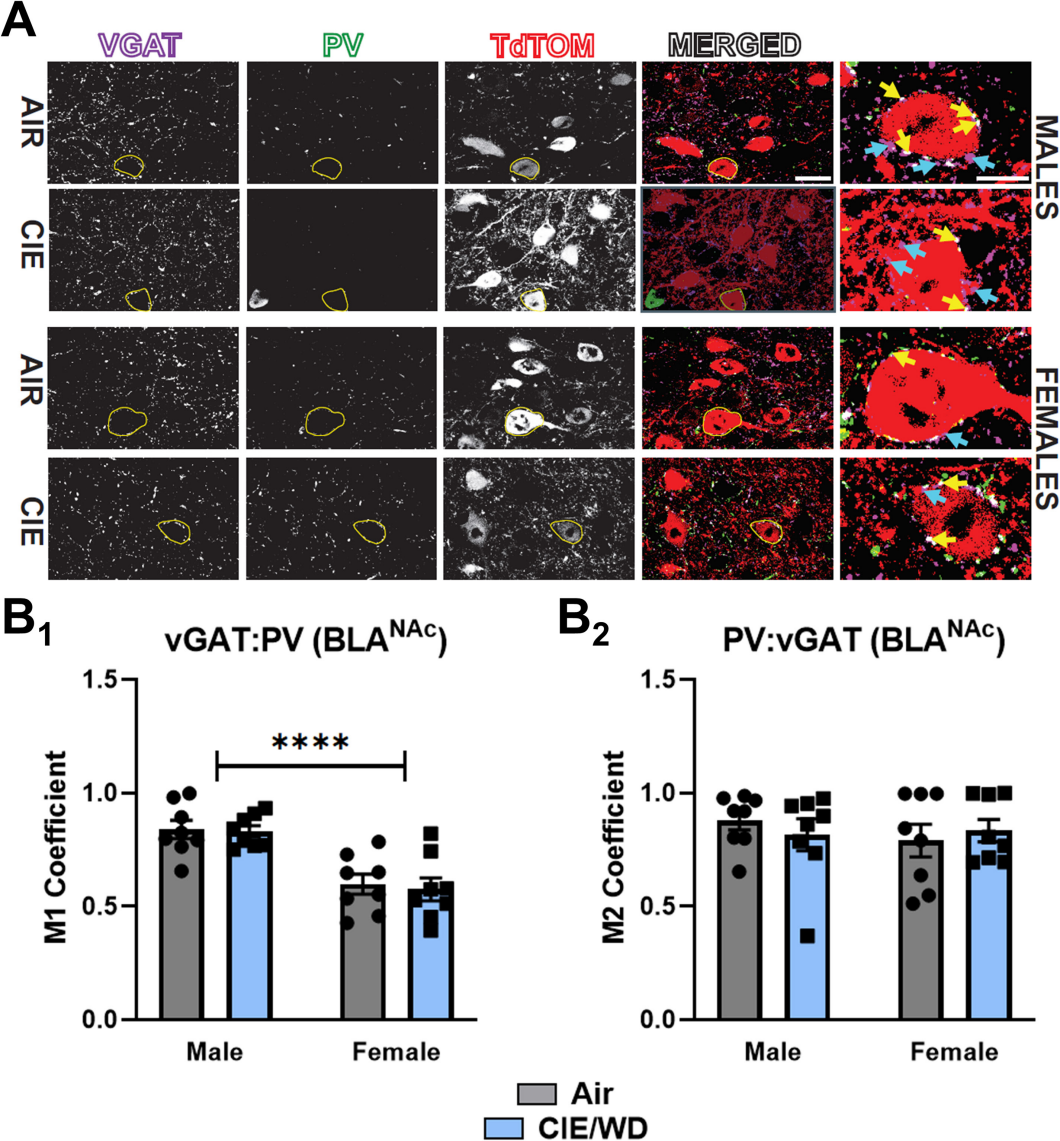
Compared to males, females have less colocalization between vGAT and PV expression associated with BLA^NAc^ neurons. (A) Representative images of vGAT (purple), PV (green), tdTomato (red) and overlap (‘Merged’) at 40× magnification in males. ROIs drawn around tdTomato^+^ soma (BLA^NAC^ neurons) are indicated across each panel and enlarged for colocalization comparisons (far right). Yellow arrows indicate vGAT^+^/PV^+^ puncta within tdTomato^+^ ROIs. Blue arrows indicate apparent vGAT^+^/PV^−^ puncta within these regions. (B) Manders overlap values for vGAT and PV staining in air‐ and CIE/WD‐exposed male and female BLA^NAC^ neurons. For M1 (B_1_), two‐way ANOVA across sex and treatment factors revealed no interaction but significantly less overlap of vGAT^+^ puncta with PV^+^ puncta in females relative to males regardless of treatment; sex: *p* < 0.0001, *F*(1, 28) = 35.92, *n* = 8 slices per condition from three different animals. For M2, there were no significant interaction or main effects of either sex or CIE/WD for the colocalization of PV^+^ puncta with vGAT^+^ puncta.

In the BLA, local inhibitory synapses innervate perisomatic regions of principal neurons largely arising from PV‐containing interneurons [23, 42, 43, 44, 45]. Given the greater apparent GABA release from local interneurons in females, we analysed immunostaining of PV and the presynaptic vesicular GABA transporter (vGAT) along with tdTomato^+^ somata to highlight GABAergic inputs onto BLA^NAC^ principal neurons. We then used confocal microscopy and quantified colocalization of these markers within ROIs represented by tdTomato^+^‐labelled BLA^NAC^ somata (Figure 5A). There were no interactions or main effects of either sex or CIE/WD on the relative puncta intensity for both vGAT, interaction *F*(1, 28) = 0.1634, *p* = 0.689; sex *F*(1, 28) = 1.259, *p* = 0.271; CIE/WD *F*(1, 28) = 1.58 × 10^−5^, *p* = 0.997, and PV, interaction *F*(1, 28) = 0.202, *p* = 0.657; sex *F*(1, 28) = 0.777, *p* = 0.386; CIE/WD *F*(1, 28) = 0.128, *p* = 0.724. We employed Mander's overlap analysis [35] to quantify the colocalization between PV^+^ and vGAT^+^ puncta across conditions (white puncta, Figure 5A). For the overlap between vGAT with PV near BLA^NAC^ somata, there were no significant interactions between sex and CIE/WD, *F*(1, 28) = 0.029, *p* = 0.866, two‐way ANOVA, or main effect of CIE/WD, *F*(1, 28) = 0.1365, *p* = 0.715. However, there was a significant main effect of sex, *F*(1, 28) = 35.92, ****p < 0.0001 (Figure 5B_1_), with vGAT:PV colocalization being lower in females relative to males. Conversely, PV colocalization with vGAT (Figure 5B_2_) showed no significant main effects of either CIE/WD, *F*(1, 28) = 0.026, *p* = 0.872, or sex, *F*(1, 28) = 0.338, *p* = 0.566, and no significant interaction between these factors, *F*(1, 28) = 0.797, *p* = 0.380. This suggests that, despite higher apparent GABA release probability from female local inhibitory synapses, these synapses may be represented by fewer PV^+^ GABA synapses relative to those arising from other types of interneurons.

### 3.5 CIE/WD Reduced Spontaneous GABA Release Onto BLA^BNST^ Neurons

**FIGURE 6:**
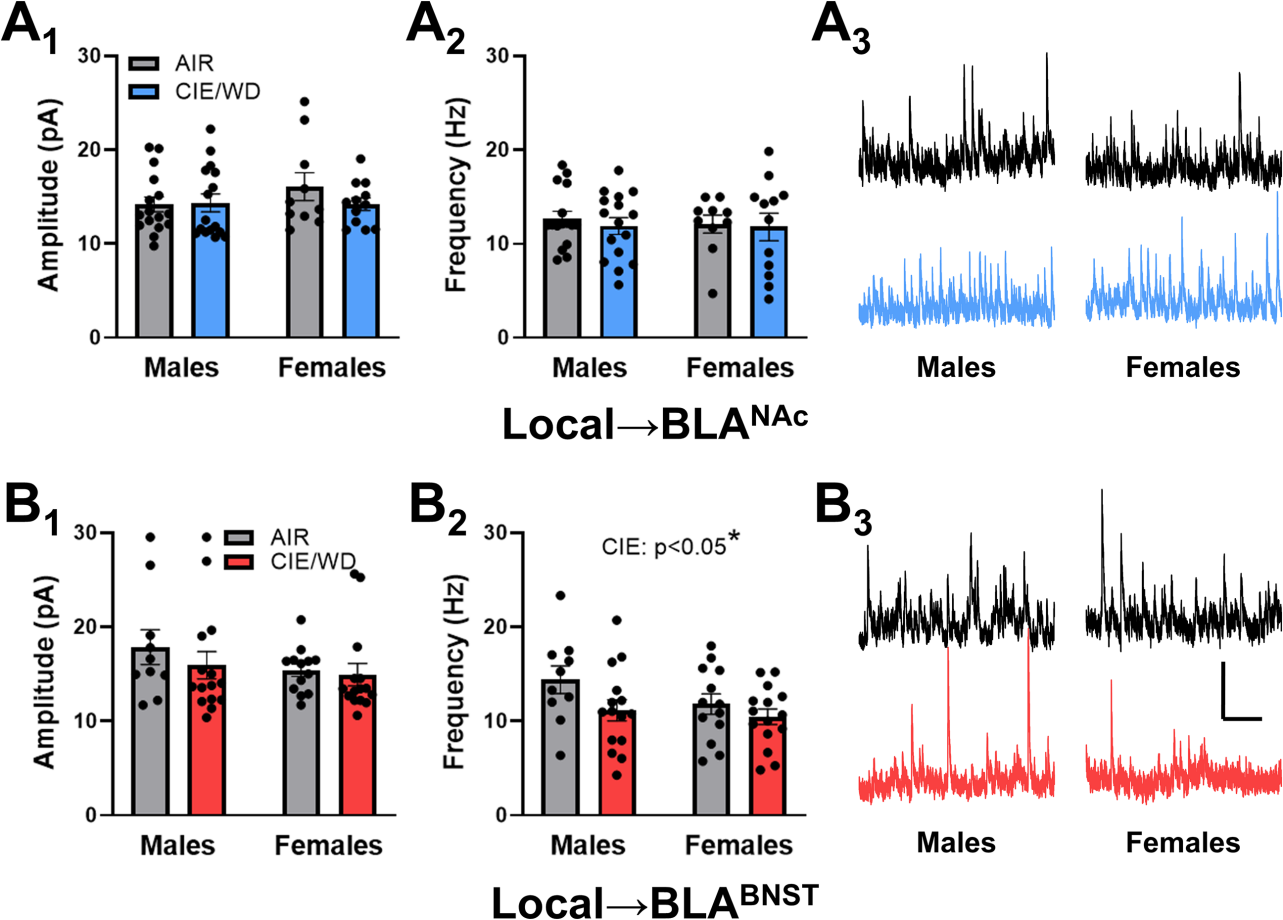
CIE/WD reduces spontaneous GABA release onto BLA^BNST^ neurons*.* (A) sIPSCs recorded from BLA^NAC^ neurons. A two‐way ANOVA found no significant effects on sIPSC amplitude (A_1_) or frequency (A_2_) in BLA^NAC^ neurons from either sex (males *N* = 16 AIR, 16 CIE/WD, females *N* = 10 AIR, 12 CIE/WD). Representative traces of sIPSCs in BLA^NAC^ neurons (A_3_). (B) sIPSCs recorded from BLA^BNST^ neurons. CIE/WD did not alter sIPSC amplitude (B_1_) but significantly reduced sIPSC frequency (B_2_) regardless of sex (two‐way ANOVA, main effect of CIE/WD, *p* < 0.05*), indicating a decrease in spontaneous GABA release onto BLA^BNST^ neurons (males *N* = 10 AIR, 15 CIE/WD; females *N* = 13 AIR, 15 CIE/WD). Representative traces of sIPSCs in BLA^BNST^ neurons (B_3_).

Given the elevated levels of GABA release from female local GABA synapses and the lower representation of PV^+^ within these inputs, we measured sIPSCs from BLA^NAC^ and BLA^BNST^ neurons. In the BLA of alcohol‐naïve animals, sIPSCs primarily reflect the intrinsic activity of ‘local’ interneuron synapses rather than LPCs given their unique pharmacology [33] and their dominant perisomatic innervation of BLA principal neurons [44]. The amplitude of sIPSCs can reflect postsynaptic GABAergic function, while their frequency can reflect GABA release probability, the number of local GABA synapses or the excitability of these interneurons [46, 47, 48]. In BLA^NAC^ neurons (Figure 6A), there were no significant effects of either CIE/WD or sex on sIPSC amplitude or frequency. In BLA^BNST^ neurons (Figure 6B), we found no effect of sex or CIE/WD on sIPSC amplitude. However, CIE/WD significantly decreased sIPSC frequency in this population of principal neurons, *F*(1, 49) = 4.176, *p = 0.046, regardless of sex, main effect, *F*(1, 49) = 2.119, *p* = 0.152; interaction, *F*(1, 49) = 0.704, *p* = 0.405.

## 4 Discussion

Our findings demonstrate that CIE/WD has selective effects on the GABAergic system in the BLA that are dependent on the interneuron population, projection target of the BLA principal neuron and sex. For example, CIE/WD suppressed evoked GABA release from LPCs specifically onto male BLA^NAC^ neurons but did not impact LPC GABA release onto either female BLA^NAC^ neurons or BLA^BNST^ neurons from both sexes. The μ opioid receptor antagonist CTOP reversed this decrease in release, identifying a potential mechanism for the regulation of feed‐forward inhibition onto some BLA principal neuron populations. In contrast, evoked ‘local’ GABA release was higher onto female principal neurons relative to males, but there were no sex differences in spontaneous GABA release onto either BLA^NAC^ or BLA^BNST^ neurons in air‐exposed controls. Immunostaining revealed lower colocalization of vGAT with PV near female BLA^NAC^ neuronal somata. CIE/WD also reduced the frequency of spontaneous GABAergic neurotransmission onto BLA^BNST^ neurons but did not alter electrically evoked ‘local’ GABA release onto these neurons or BLA^NAC^ cells. Thus, sex modulates the basal characteristics of local GABA interneuron synapses, while CIE/WD drives unique adaptations in GABA neurotransmission that are dependent on sex, the origin of the GABA synapse and BLA principal neuron population.

### 4.1 LPCs

#### 4.1.1 CIE/WD Reduces GABA Release From LPCs Onto Male BLA^NAC^ Neurons

CIE/WD significantly reduces GABA release from LPCs onto BLA^NAC^ neurons in male rats but not females. Conversely, acute ethanol facilitates LPC GABAergic synaptic responses via a postsynaptic beta‐adrenergic receptor‐dependent mechanism [49]. CIE/WD exposure does not attenuate this acute facilitation [24]. Male BLA^NAC^ neurons are also more vulnerable to the CIE/WD‐mediated increase in postsynaptic glutamatergic function and neuronal excitability compared to females [5]. Together, these neurophysiological changes should increase the activity of male BLA^NAC^ neurons, leading to greater BLA output to the NAC core in male rats. The BLA‐NAC projection promotes the consumption of rewarding substances like sucrose, the reinstatement of cocaine‐seeking and positive reinforcement in intracranial self‐stimulation tasks [7, 8]. Moreover, the NAC core itself facilitates the reinstatement of alcohol‐seeking, ethanol‐conditioned place preference and binge drinking [9, 10, 11]. This suggests that the reduced LPC GABAergic function, increased glutamatergic function and increased neuronal excitability in male BLA^NAC^ neurons may be responsible for CIE/WD increasing ethanol consumption in male rats [30]. In contrast, CIE/WD does not impact GABAergic function, glutamatergic function or neuronal excitability in female BLA^NAC^ neurons [5] and has no effect on ethanol consumption in female rats [30].

#### 4.1.2 μ Opioid Receptors and CIE/WD–Induced Decrease in LPC GABA Release

LPCs, also known as the lateral intercalated cells, receive glutamatergic inputs from lateral cortical structures through the external capsule and provide feed‐forward inhibition onto the distal dendrites of glutamatergic principal neurons in the BLA [18]. Although they do not express markers typically found in cortical and ‘local’ BLA interneurons like calbindin, calretinin, PV, neuropeptide Y, cholecystokinin or somatostatin, LPCs express μ opioid receptors and receive input from enkephalin‐immunoreactive terminals [20, 40]. Previous studies examining the related medial intercalated cells have shown that acute methionine‐enkephalin exposure reduced feed‐forward GABAergic inhibition onto downstream neurons in the central amygdala [50]. Similarly, the μ receptor agonist DAMGO hyperpolarizes medial intercalated cells but has no effect on the resting membrane potential of glutamatergic principal neurons in the BLA, which may illustrate a cellular mechanism reducing GABAergic feed‐forward inhibition into the central amygdala [41]. We show here that DAMGO also dramatically reduces LPC feed‐forward GABAergic transmission onto BLA principal neurons and that the μ receptor antagonist CTOP can reverse the CIE/WD‐dependent suppression of LPC GABA release onto male BLA^NAC^ neurons. This strongly argues that CIE/WD facilitates μ receptor signalling/function or expression in LPC neurons. This may represent a compensatory downregulation of these synapses, albeit through an independent mechanism, in the face of continuous facilitation by acute ethanol. The specific mechanism linking acute ethanol facilitation of GABA release via beta adrenergic receptors [49] and the μ opioid receptor dependent downregulation of LPC GABAergic function following chronic ethanol remains to be determined. It is also unclear why this μ‐mediated inhibition of LPC GABA release might be both sex‐ and principal neuron population–specific. Inhibition of the peptidase neprilysin dramatically facilitates enkephalin modulation of extrinsic glutamate inputs onto medial intercalated GABAergic neurons [51]; and neprilysin mRNA levels are decreased by ethanol exposure in some preclinical models [52]. Population‐ and sex‐specific downregulation of neprilysin activity/expression following CIE/WD is thus an attractive candidate mechanism that may warrant further investigation.

### 4.2 Local Interneurons

#### 4.2.1 Basal Sex Differences in Evoked GABA Release From Local Interneurons

Although CIE/WD did not affect evoked GABA release from local interneurons, our data suggest that female local interneurons release more GABA onto both BLA^NAC^ and BLA^BNST^ neurons compared to males. Local interneurons in the BLA are exceptionally diverse and can express a variety of protein markers, including PV, calbindin, calretinin, neuropeptide Y, cholecystokinin and somatostatin [18, 21, 22, 23]. Local interneurons expressing PV or calbindin represent the most abundant subtypes, although they are partially overlapping populations [21, 23]. Regardless, PV terminals form dense perisomatic baskets around BLA principal neurons [42] making PV‐expressing interneurons the most likely source for greater evoked ‘local’ GABA release onto female BLA^NAC^ and BLA^BNST^ cells. Female non‐human primate BLA tissue expresses greater amounts of the vesicle priming protein Munc13‐1 [53], which could indicate that the increased release from local GABA synapses in female rats may be related to a larger pool of primed vesicles. Low levels of oestrogen and progesterone during dioestrus also enhance the function of female PV^+^ neuron GABA synapses [26]. Indeed, we previously showed that, relative to air‐treated controls, the 10‐day CIE/WD treatment enhances the proportion of females found to be in dioestrus [28]. This also implicates CIE/WD‐dependent hormonal disruptions as a potential mechanism. Importantly, our vGAT and PV immunostaining strongly suggests that, relative to males, female perisomatic inhibitory synapses may include inputs from a greater diversity of PV^−^ interneurons. Male‐ and female‐specific interneuron populations might also explain the sex‐dependent expression of apparent GABA release from local synapses following electrical stimulation. Since sIPSCs represent perisomatic GABA synapses and sIPSC frequencies were similar between males and females in air‐exposed controls, these sex differences likely represent distinct release probabilities by synapses arising from unique populations of interneurons. Given that local interneurons primarily provide feedback inhibition onto glutamatergic principal neurons in the BLA [45, 54, 55], the sex differences in evoked local GABA release may help regulate distinct neurophysiological outcomes following chronic ethanol.

#### 4.2.2 CIE/WD Reduces Spontaneous GABA Release Onto BLA^BNST^ Neurons

In alcohol‐naïve animals, spontaneous GABA release in the BLA primarily reflects activity from local interneurons [33, 44]. However, some forms of chronic stress can remodel BLA principal neuron dendritic morphology [56, 57]. Given the potential neurobiological similarities and interactions between chronic stress and ethanol [58], additional GABAergic cell types including the LPCs might contribute to the synapses giving rise to perisomatic sIPSCs following CIE/WD. Regardless, our findings indicate that CIE/WD reduced the frequency of spontaneous GABA release onto BLA^BNST^ neurons regardless of sex. Spontaneous inhibitory neurotransmission can suppress action potential firing [59]. Since the BNST regulates anxiety‐like behaviour [14, 15, 16], CIE/WD disruption of spontaneous GABA release onto BLA^BNST^ neurons could thus enhance their activity and promote their contribution to withdrawal anxiety‐like behaviour. Given the absence of a CIE/WD effect on either local GABA release (Figure 4) or the distribution of BLA GABAergic markers (Figure 5), these findings suggest that CIE/WD may alter the intrinsic excitability of local interneurons projecting onto BLA^BNST^ principal cells or may modify the direct interactions between local interneurons [43, 60]. Nicotinic receptors expressed by BLA interneurons positively influence their excitability [61]; and we have recently shown that CIE/WD dysregulates the function of BLA cholinergic inputs from the basal forebrain [29]. BLA interneurons also express presynaptic kainate‐type glutamate receptors that bidirectionally modulate terminal excitability with modest activation enhancing GABA release and robust activation inhibiting it [62]. Importantly, CIE/WD enhances presynaptic glutamate release onto BLA^BNST^ neurons from both sexes [5]. These latter observations combined might explain the inhibitory effects of CIE/WD on sIPSC frequency within a specific population of neurons. Alternatively, BLA interneurons can potently influence one another via GABAergic synapses and electrical coupling [60, 63]. CIE/WD dysregulation of distinct interneuron populations might disinhibit BLA principal neurons via the overt influence of local GABAergic synapses.

## 5 Conclusion

In summary, the current study demonstrated that CIE/WD and sex influence GABAergic inhibition in the BLA. CIE/WD reduced GABA release probability from LPCs onto male BLA^NAC^ neurons, which is regulated by an LPC MOR‐dependent mechanism. CIE/WD also decreased the sIPSC frequency in BLA^BNST^ neurons from both sexes, which occurs without any CIE/WD‐mediated changes to GABA release probability from local interneurons. These data suggest that CIE/WD reduces the intrinsic excitability of local interneurons or reduces the number of local interneuron inputs to BLA^BNST^ neurons. Finally, we discovered basal sex differences in GABA release probability from local interneurons such that local interneurons release more GABA onto BLA^NAC^ and BLA^BNST^ neurons from female rats compared to males. This occurred despite lower colocalization between vGAT^+^ and PV^+^ immunoreactivity and no sex differences in sIPSC frequency. These data are thus consistent with greater function or GABA release by female local interneurons compared to males. Collectively, our findings strongly suggest that CIE/WD reduces GABAergic inhibition onto BLA principal neurons in an input‐, population‐ and sex‐specific manner. These neurophysiological characteristics are poised to govern both sex‐dependent and sex‐independent effects of alcohol dependence.

## Data Availability

The data that support the findings of this study are available on request from the corresponding author. The data are not publicly available due to privacy or ethical restrictions.
